# Determining the role of IL-4 induced neuroinflammation in microglial activity and amyloid-β using BV2 microglial cells and APP/PS1 transgenic mice

**DOI:** 10.1186/s12974-015-0243-6

**Published:** 2015-03-04

**Authors:** Clare H Latta, Tiffany L Sudduth, Erica M Weekman, Holly M Brothers, Erin L Abner, Gabriel J Popa, Michael D Mendenhall, Floracita Gonzalez-Oregon, Kaitlyn Braun, Donna M Wilcock

**Affiliations:** Department of Physiology, University of Kentucky, Sanders-Brown Center on Aging, Lexington, KY 40536 USA; Department of Physiology, University of Kentucky, Lexington, KY 40536 USA; Department of Epidemiology, University of Kentucky, Lexington, KY 40536 USA; Department of Molecular and Cellular Biochemistry, University of Kentucky, Lexington, KY 40536 USA; Department of Biology, The University of Manchester, Manchester, M13 9PL UK; University of Kentucky, Room 424 Sanders-Brown Center on Aging, 800 S Limestone Street, Lexington, KY 40536 USA

**Keywords:** Inflammation, Alzheimer’s disease, Microglia, Beta-amyloid

## Abstract

**Background:**

Microglia are considered the resident immune cells of the central nervous system (CNS). In response to harmful stimuli, an inflammatory reaction ensues in which microglia are activated in a sequenced spectrum of pro- and antiinflammatory phenotypes that are akin to the well-characterized polarization states of peripheral macrophages. A “classically” activated M1 phenotype is known to eradicate toxicity. The transition to an “alternatively” activated M2 phenotype encompasses neuroprotection and repair. In recent years, inflammation has been considered an accompanying pathology in response to the accumulation of extracellular amyloid-β (Aβ) in Alzheimer’s disease (AD). This study aimed to drive an M2a-biased immune phenotype with IL-4 *in vitro* and *in vivo* and to determine the subsequent effects on microglial activation and Aβ pathology.

**Methods:**

*In vitro*, exogenous IL-4 was applied to BV2 microglial cell cultures to evaluate the temporal progression of microglial responses. *In vivo*, intracranial injections of an adeno-associate-virus (AAV) viral vector were performed to assess long-term expression of IL-4 in the frontal cortex and hippocampus of Aβ-depositing, APP/PS1 transgenic mice. Quantitative real-time PCR was used to assess the fold change in expression of biomarkers representing each of the microglial phenotypes in both the animal tissue and the BV2 cells. ELISAs quantified IL-4 expression and Aβ levels. Histological staining permitted quantification of microglial and astrocytic activity.

**Results:**

Both *in vitro* and *in vivo* models showed an enhanced M2a phenotype, and the *in vivo* model revealed a trend toward a decreased trend in Aβ deposition.

**Conclusions:**

In summary, this study offers insight into the therapeutic potential of microglial immune response in AD.

## Introduction

Alzheimer’s disease (AD) was first identified in 1907 by Alois Alzheimer when investigating the case of a female patient, Auguste D, suffering from irrational personality changes and severe memory impairment. Post mortem analysis revealed a severely atrophied brain, and histological staining revealed amyloid-β (Aβ) plaques and neurofibrillary tangles composed of hyperphosphorylated and aggregated tau. In addition, Alzheimer noted a proliferated cluster of immune regulatory and support cells surrounding the Aβ plaques and termed this “*gliose*”, now known as the inflammatory response “gliosis” [1]. This observation signified a potential interaction between immune regulation and the development Aβ lesions, a critical relationship that had previously been understudied until the last 2 decades [2]. Inflammation, the final pathology of the triad, marks the body’s protective response to detrimental stimuli and injury. To date, an abundance of studies has confirmed the role of inflammation in the pathological progression of AD [3].

Derived from a mesodermal myeloid origin, microglia act in parallel to their sister immune regulatory cells of the periphery, macrophages, in the central nervous system (CNS) [4]. Current understanding of microglial physiology and activity, including phenotypic diversity, has been extrapolated. In response to immune stimulation, a spectrum of macrophage phenotypes is unveiled [5,6]. M1 activation, or classical activation, is considered the first line of defence to eradicate invading pathogens and is both driven and characterized by the release of proinflammatory cytokines such as interleukin 1β (IL1β) and tumor necrosis factor α (TNFα). On the other side of the spectrum, M2 activation, or alternative activation, is associated with wound repair and healing, as well as the ability to dampen the M1 response [7-10]. M2 activation is broken into additional sub-categories. M2a activation is characterized by the expression of mannose receptor MRC1 and extracellular matrix modeling ability with the expression of chitinase 3-like 3 proteins (YM1 in mice CHL3 in humans) and arginase 1, ARG1. Sequentially, acquired “deactivation” of the immune response is achieved by an M2c phenotype upon interleukin 10 (IL-10) exposure with amplified expression of transforming growth factor β1 (TGFβ1) and sphingosine kinase 1 (SPHK1). The role of an M2b phenotype is relatively unknown. Nonetheless, it has been defined by an elevated expression of the CD86 and major histocompatibility complex II (MHCII) receptors in comparison to the low constitutive expression of these receptors across all activation phenotypes [7,10].

An intricate cytokine network tightly controls the amplification and reduction of each of the microglial states. IL-4 is considered to be the strongest polarizing cytokine toward an M2a response [11]. IL-13 has also been noted as an M2a polarizing cytokine. The interaction of an M2a phenotype with Aβ remains unclear. The induction of an M2a phenotype via IL-4 exposure has indicated both attenuation and amelioration of the neurotoxic peptide depositions in conflicting *in vivo* studies [12,13].

To assess M2a-driven modulation of microglial activation, IL-4 was endogenously applied to BV2 microglial cells and temporal changes in phenotype and morphology were monitored. Results revealed a robust M2a phenotype peaking at 8 h for the majority of phenotypic markers. Amplification of alternative phenotypic genes also revealed a peak in expression suggesting a minutely heterogeneous phenotype. Morphology showed reduced extended process upon IL-4 application, with the effects dampened over time and extended process formation. In order to better understand the effect of an M2a phenotype on Aβ deposition, bilateral injections of an adeno-associated virus (AAV) expressing IL-4 were inserted into the frontal cortex and hippocampus of APP/PS1sw mice. A robust M2a phenotype was successfully induced with the AAV IL-4 injection. Histological staining revealing increased microgliosis and astrogliosis in the AAV-IL-4 injected mice. Lastly, Aβ levels measured biochemically were moderately reduced in comparison to the mice treated with AAV-GFP, therefore providing insight into a potential mechanism of ameliorating Aβ pathology in AD.

## Material and methods

### BV2 cell culture

BV2 cells were obtained from Dr. Linda Van Eldik, University of Kentucky, Lexington, KY. Cells were grown on six-well plates in FX12 media containing 10% fetal bovine serum, 1% serum L-glutamate and 1% streptomycin (Life Technologies, Carlsbad, CA) until 80% confluency was obtained approximately 3 days after passage. Cells were starved of serum for 24 h prior to treatment. Cells were stimulated with an exogenous application of murine IL-4 (20 ng/ml; R & D Systems, Minneapolis, MN) in the serum-free medium added to the culture. Serum-free medium was applied as the no treatment control. Cells were incubated (5% CO_2_, 37°C) for 2, 4, 6, 8, 10, 16 and 24 h before removal. Media were aspirated, and cells were rinsed twice in Dulbecco’s phosphate-buffered saline pH 7.4 (DPBS) (Life Technologies, Carlsbad, CA) at a 1× dilution for 5 min and frozen at −80°C. Cell culture experiments were performed in triplicate and repeated at least three times at different passage numbers.

### Immunofluorescent staining

BV2 cells were grown on glass coverslips in 12-well plates until 80% confluency after approximately 3 days from passage. Twenty-four hours prior to the experiment, cells were starved and treated as above. After incubation cells were rinsed in 1× DPBS, transferred to a clean 12-well plate, fixed in 10% neutral buffered formalin (Sigma-Aldrich, St Louis, MO) for 20 min and rinsed twice in 1× DPBS for 5 min. Cells were blocked in 0.019% L-lysine, 0.3% triton-X (Sigma-Aldrich, St Louis, MO) and 4% goat serum (Pel-Freez, Rogers, AR) in DPBS for 45 min and incubated in primary antibody CD11b 1:500 (Rat monoclonal, AbD Serotec, Raleigh, NC) in 1× DPBS overnight at 4°C. Cells were left to acclimate to room temperature for 1 h and rinsed twice in 1× DPBS for 5 min before incubation in secondary goat anti-rat (488 nm) at a 1:20,000 dilution of for 1 h. Glass coverslips were rinsed twice in 1× DPBS prior to adhesion to microscope slides using Permafluor mountant (Thermo Scientific, Fremont, CA). Fluorescence was visualized using Nikon Elements BR image analysis system (Melville, NY) at 60× magnification. The same settings were applied to all of the images.

### Animals

This study was approved by the University of Kentucky Institutional Animal Care and Use Committee and conformed to the National Institutes of Health Guide for the Care and Use of Animals in Research. Fourteen APP/PS1 transgenic mice [14], a C57BL6 strain of mice with human APPSwe and PS1-dE9 mutations, were bred in the Division of Laboratory Animal Resources (DLAR) at the University of Kentucky and aged for 3 months prior to surgery. The animals were randomly assigned into one of two groups for adeno-associated virus type 8 (AAV-8) injection, either the AAV-GFP control group (*N* = 8) or the AAV-IL-4 group (*N* = 6). Data revealed no gender-dependent differences and were therefore analyzed together. Mice were killed 43 days post-surgery because of increased deaths in the IL-4-AAV-injected animals.

### AAV preparation

The vector for preparing recombinant AAV was constructed by ligating the 1,349-bp *Eco*RI/*Sal*I fragment carrying the IRES-GFP from pSMPUW-IRES-GFP (Cell Biolabs) into *Eco*RI/*Sal*I-digested pZac2.1 (gift of Dr. Paul Murphy, University of Kentucky) to create ViCo1.28. The IL-4 insert was prepared by polymerase chain reaction (PCR) amplification of cDNA clones obtained from Open Biosystems (GE Healthcare, Dharmacon RNAi and gene expression, Piscataway, NJ). The IL-4 PCR primer sequence used was ‘CCCGCTAGCGACGGCACAGAGCTATTG.

CCACCGCGGGGCTCAGTACTACGAGTA’ (Open Biosystems, GE Healthcare, Lafayette, CO; Cat. no.: MMM1013-7510313, clone ID 3376587 and accession BC027514). The primers introduced a *Nhe*I site at the 5′-end and a *Sac*II site at the 3′-end of each cytokine gene to facilitate cloning into the corresponding sites in ViCo1.28. The fidelity of each clone was confirmed by DNA sequence analysis.

AAV-8 coat protein-pseudotyped AAV-2 viruses were prepared by co-transfecting 10 T225 culture flasks of 293LTV cells (Cell Biolabs) with 250 mg pAAV2/8 (obtained from the University of Pennsylvania Viral Core), 500 mg pAdDF6 (gift of Dr. Paul Murphy, University of Kentucky) and, individually, 250 mg of each cytokine clone using 5 mg polyethyleneimine to enhance DNA uptake. After 3 days, the cells were harvested, washed, suspended in 13 ml 150 mM NaCl, 50 mM Tris · Cl pH 8.4, 0.5% deoxycholate and 50 U/ml of benzonase and incubated at 37°C for 30 min. An additional 2.8 ml 5 M NaCl was added, and the incubation was continued for another 30 min at 45°C. The cell suspension was then subjected to four freeze/thaw cycles (30 min at −80°C/ 30 min at 45°C). The lysate was then partially clarified by centrifugation at 18,500 × g for 10 min at 20°C. The supernatant was laid on top of an iodixanol step gradient and centrifuged at 350,000 × g for 1 h at 18°C. The interface between the 40% and 54% iodixanol layers was withdrawn and spin-purified and concentrated using four washes with PBS in an Amicon Ultra-15 100,000 MWCO spin concentrator. The virus preparation was then titered using real-time PCR with primers directed against the CMV promoter region of the DNA encapsulated in the virions.

### Bilateral intracranial injections

**Figure 1:**
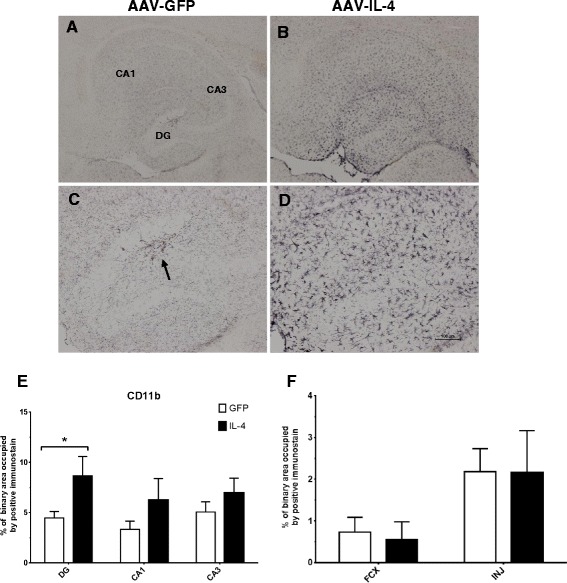
**IL-4 shows increased CD11b immunostaining. A** and **B** CD11b staining of hippocampal sections of the AAV-GFP- and the AAV-IL-4-injected mice, respectively. **C** and **D** Staining in the dentate gyrus (*DG*); an *arrow* shows the site of the hippocampal injection. **E** The percentage of binary area occupied by the positive immunostain in the hippocampus across all samples; **F** uses (*N* = 4) for AAV-IL4 samples because of damage in the frontal cortex of two AAV-IL-4 mice. *P < 0.05 between the AAV-GFP control (*N* = 8) and AAV-IL-4 (*N* = 6).

Mice were anesthetized with 5% isoflurane (Butler-Schein, Dublin OH) with oxygen levels kept at 1 L/minute and immobilized in stereotaxic apparatus. They were then maintained for the duration of surgery between 1% and 2% isoflurane. The skin was shaved and sterilized with a series of iodine and ethanol treatments before the cranium was exposed by a mid-sagittal incision. Four burr holes were made over the frontal cortex and hippocampus of both the left and right hemispheres using a dental drill mounted onto a stereotaxic frame. The coordinates for injection were confirmed by our laboratory prior to the study using a dye to visualize placement. Using bregma as the origin, burr holes were made in the frontal cortex (+2.0 mm anteroposterior, ±2.0 mm lateral) and in the hippocampi (−2.7 mm anteroposterior, ±2.5 mm lateral) (as shown in Figure 1). At each of these sites, a 10-μl Hamilton syringe fitted with a 26-guage needle was lowered to −3.0 mm ventral to bregma (Hamilton, Reno, NV). Then 2 μl of either AAV-GFP or AAV-IL-4 at a concentration of 5× 10^10^ genomes/μl was administered over a 2-min period at each injection site. The mid-sagittal incision was cleaned and sutured, and a 4% lidocaine ointment was applied. Animals were group housed, based on sex, under standard vivarium conditions. Sutures were removed 10 days post-surgery.

### Tissue processing

After 43 days post-surgery, the mice were administered a lethal intraperitoneal injection of 0.5 ml of Buthanasia-D (Butler-Schein, Dublin OH) and intracardially perfused with 25 ml 0.9% saline (Ricca Chemical Co., Arlington, TX). The brain was removed immediately and bisected along the mid-sagittal plane. The left hemisphere was post-fixed in 4% paraformaldehyde for 24 h before being treated for cyroprotection through a series of 10, 20 and 30% sucrose concentrations each for an additional 24 h. The left hemisphere was placed on a frozen stage and sectioned into 25-μm horizontal sections, which were collected using a sliding microtome. The sections were stored free-floating in 1× DPBS plus sodium azide at 4°C. Sections were stored in 24-well plates at 4°C until required. The frontal cortex and hippocampus of the right hemisphere were micro-dissected, snap-frozen in liquid nitrogen and stowed at −80°C.

### Immunohistochemistry

A Nissl stain was used to determine the injection site location determined as previously described [15]. Eight free-floating 25-μm sections spaced ~400 μm from each other were collected 1,300-3,400 μm ventral to bregma and separated into 1× DPBS. Initially, the free floating sections were permeabilized, and endogenous peroxidase sites were inhibited via incubation with 10% methanol and 3% hydrogen peroxide in 1× DPBS for 15 min. Sections were blocked with 0.3% triton-X, 4% goat serum and 0.019% L-lysine in 1× DPBS. Sections were incubated in primary antibodies CD11b, 1:3,000 (Rat monoclonal, AbD Serotec, Raleigh, NC) and GFAP, 1:10,000, (Rabbit polyclonal, Dako, Denmark) with 4% goat serum in DPBS left at room temperature and then overnight at 4°C. After incubation and three 5-min washes in 1× DPBS, sections were incubated in biotinylated secondary antibodies; goat anti-rabbit IgG was used for GFAP and goat anti-rat for CD11b, both at 1:3,000 (Vector Laboratories, Burlingame, CA) for 2 h. Amplification of the secondary antibody signal was achieved through incubation in advidin-biotin complex (ABC) (Vector Laboratories, Burlingame, CA) for 1 h. For color development the vector diaminobenzidine (DAB) peroxidase kit (Vector Laboratories, Burlingame, CA) was used according to the manufacturer’s instructions. Sections were mounted onto slides, left to air-dry overnight, dehydrated in an ethanol gradient followed by xylene incubations and cover-slipped using DPX mountant (Electron Microscopy Sciences, Hatfield, PA).

Images of the stained sections were taken at 200× magnification using the Nikon Elements BR image analysis system (Melville, NY) in the frontal cortex (FCX). Hippocampal fields were localized to the dentate gyrus (DG), cornu ammonis 1 (CA1) and cornu ammonis 3 (CA3) and images collected at the same magnification. Anatomical structures in the tissue were used to ensure uniformity in the image. Microscope settings were saved and were the same for all of the images in each stain. A threshold for positive immunostaining was set, saved and applied to all of the images of a given stain. The data yielded the percentage of binary area occupied by the positive immunostain. Approximately six sections per mouse were used for analysis in the hippocampus and the frontal cortex. Tissue damage in the frontal cortex of several animals left large holes interfering with quantification of the positive immunostain analysis. Therefore, two mice in the AAV-IL-4 group were exempt from this analysis.

### ELISA measurements

Protein was extracted from the frontal cortex tissue by homogenization in mammalian protein extraction reagent (MPER) lysis buffer with protease and phosphatase inhibitor (Pierce Biotechnology Inc., Rockford, IL). The samples were centrifuged for 15 min at 10,000 rg at 4°C. The supernatant was collected as the soluble extract, leaving a pellet of remaining insoluble extract. A Bicinchonic Acid protein assay kit (BCA) (Pierce Biotechnology Inc., Rockford, IL) was performed to obtain the concentration of the soluble extract according to the manufacturer’s instructions. The soluble extracts were normalized to the lowest concentration using the MPER lysis buffer. Then 250 μl of 70% formic acid was added to the insoluble extract before ultracentrifugation at 47,000 rpm at 4°C for 1 h. The supernatant was collected and neutralized 1:20 with Tris–HCl (pH 11). Additional HCl was added to bring the sample accurately to pH 7 using a drop pipet. A Meso Scale Discovery (MSD) 96-Well MULTI-SPOT Human (6E10) A-beta Triplex Assay (MSD, Gaithersburg, MD) was performed on both the soluble and insoluble extractions, according to the manufacturer’s instructions, to determine the concentrations of Aβ38, Aβ40 and Aβ42. Murine IL-4 levels in the soluble extracts were detected using the the Mouse IL-4 Quantikine ELISA kit (R & D Systems Inc., Minneapolis, MN). All steps followed the manufacturer’s instructions. A second BCA was performed on soluble extracts to confirm the protein concentration, and samples were normalized prior to the ELISA measurement.

### Quantitative real-time RT-PCR

RNA was extracted from the frozen hippocampus of the right hemisphere by rotor homogenization Bio-Gen Pro200 (Pro-Scientific, Oxford, CT) in the RNA-Solv Reagent of the RNeasy tissue kit (Qiagen, Valencia, CA); the subsequent steps of the extraction were achieved according to the manufacturer’s instructions. BV2 cell homogenization RNA was extracted from the BV2 cells using cell scrapers in RLT buffer of the RNeasy Mini-kit (Qiagen, Valencia, CA). Again, the subsequent steps of the extraction were achieved according to the manufacturer’s instructions. The Biospec-Nano Spectrophotometer (Shimadzu, Kyoto, Japan) was used to quantify the nucleic acid concentration in the RNA samples. The RNA concentrations were standardized to the lowest concentration with RNAase free water. The standardized samples were added to the High Capacity RNA-to-cDNA kit (Applied Biosystems, Foster City, CA) including reverse transcriptase, random primers and buffer according to the manufacturer’s instructions. The cDNA was produced through a series of heating and annealing cycles in the Veriti™ 96-well Thermocycler (Applied Biosystems, Grand Island, NY).

Quantitative real-time RT-PCR was achieved using the Fast-TaqMan Gene Expression Kit in 96-well plates (Life Technologies, Carlsbad, CA); 0.5 μl of the cDNA (100 μl of standardized RNA samples) was diluted with 6.5 μl of RNAse-free water and added to each well; 1 μl of the selected gene probe (Life Technologies, Carlsbad, CA) listed in Table 1 was added to 10 μl of the Fast-TaqMan reagent and added to each well. Real-time RT-PCR was performed using the ViiA™7 Real Time PCR system (Applied Biosystems, Grand Island, NY). All genes measured were normalized to the constitutively expressed 18S rRNA gene. The gene expression of the AAV-IL-4 mice was compared relative to the AAV-GFP control group. Fold change was calculated using the 2^–ΔΔCt^ method [16].

**Table 1:** **Genes for RT-qPCR**

**Gene of interest**	**PMID**	**Taqman ID**
IL-1β	NM_008361.3	Mm00434228_m1
IL-12	NM_008351.2	Mm00434165_m1
TNFα	NM_013693.3	Mm00443258_m1
IL-6	NM_031168.1	Mm00446190_m1
IFNγ	NM_008337.3	Mm01168134_m1
IL1ra	NM_031167.5	Mm00446186_m1
ARG1	NM_007482.3	Mm00475988_m1
YM1 (Chil3)	NM_009892.2	Mm00657889_mH
FIZZ1	NM_020509.3	Mm00445109_m1
MRC1	NM_008625.2	Mm00485148_m1
CD86	NM_019388.3	Mm00444543_m1
FcγR1	NM_010186.5	Mm00438874_m1
FcγR3	NM_010188.5	Mm00438882_m1
TGFβ1	NM_011577.1	Mm01178820_m1
SPHK1	NM_011451.3	Mm01252544_m1

### Data analysis

For the *in vitro* analysis*,* normalized fold change values were log-transformed in order to apply parametric statistical methods. Repeated measures ANOVA, with an unstructured covariance matrix, was then used to estimate mean log fold change at each time point within each gene. The time point at which expression peaked was determined by post hoc testing such that the largest point estimate observed was then compared to the remaining time points. No ties were observed for peak time (i.e., the largest point estimate was identifiable for each gene). Repeated measures analyses were performed using the MIXED procedure in SAS 9.3® (SAS Institute, Inc., Cary, NC). Otherwise, group differences were assessed using two-sample Student’s *t*-tests. Analysis tests and data representation were performed using GraphPad Prism version 6.01 for Windows (GraphPad Software, La Jolla, CA, USA). Statistical significance was set at 0.05.

## Results

**Figure 2:**
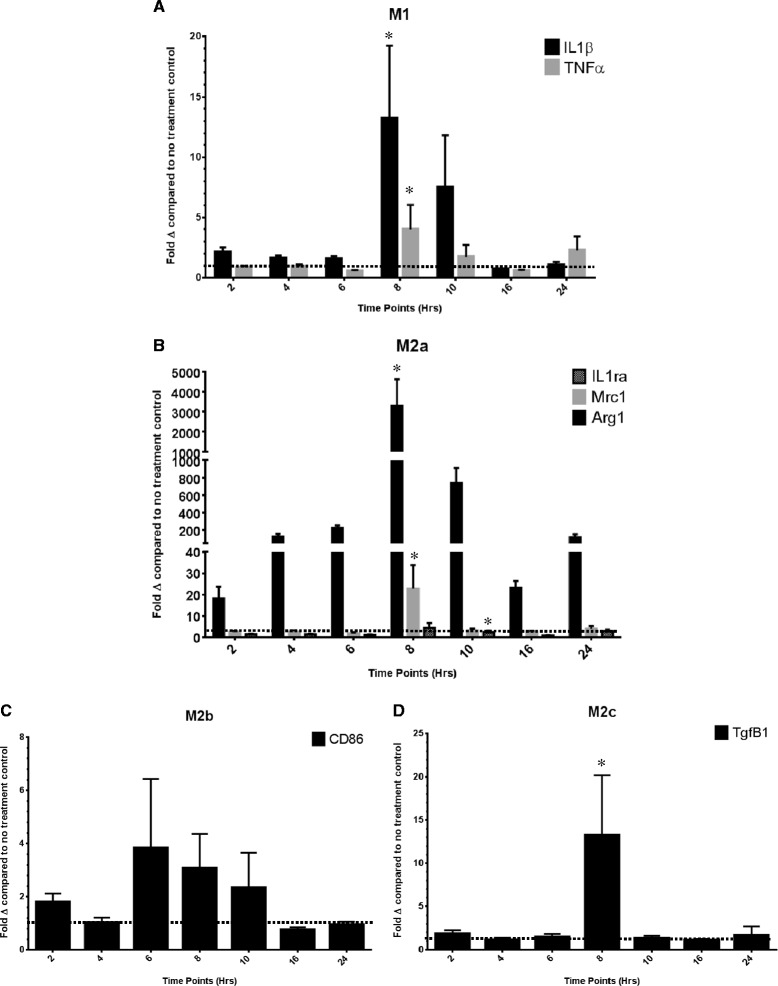
**The relative gene expression for genes representative of the (A) M1, (B) M2a, (C) M2b and (D) M2c phenotypes of the IL-4-treated cell culture.** Data are shown as the average fold change (± SEM) relative to the no treatment control at the 2-, 4-, 6-, 8-, 10-, 16- and 24-h time points. *The peak time point of gene expression determined by a repeated measures ANOVA of the fold changes in logarithmic form.

To determine the effect of the murine IL-4 application on BV2 cells, RNA was extracted, and qRT-PCR was performed on the gene markers specific to each phenotype (M1, M2a, M2b, M2c) as shown in Figure 2. Fold change was calculated relative to cell culture exposed to a no-treatment control. Both IL1β and TNFα of the M1 phenotype show a peak at 8 h (Figure 2A). In the M2a phenotype, ARG1 is largely amplified to 3,500 fold at 8 h; additionally, MRC1 also peaks at 8 h. IL1ra shows a significant peak at 10 h post treatment (Figure 2B). YM1 was found to have aberrant expression in both the control and IL-4-treated cells and was therefore exempt from the analysis. No significant differences can be seen in the M2b phenotype over time (Figure 2C), but we found elevated levels of Tgfβ1 in the M2c phenotype (Figure 2D). Table 2 shows the individual *P* values for the comparisons of gene expression over the time course.

**Table 2:** ***P*** **values for individual time-point comparisons for qRT-PCR in IL-4-treated BV2 cell culture**

**IL1β**		**2**	**4**	**6**	**8**	**10**	**16**	**24**
	**2**		0.9613	0.3623	0.0024	0.3746	0.0354	0.0218
	**4**			0.4365	0.0270	0.2481	0.0405	0.168
	**6**				0.0122	0.2677	0.0698	0.1067
	**8**					0.4261	0.0024	0.0006
	**10**						0.0792	0.0520
	**16**							0.8212
	**24**							
**TNFα**		**2**	**4**	**6**	**8**	**10**	**16**	**24**
	**2**		0.2953	0.0987	0.0252	0.9023	0.6955	0.5087
	**4**			0.0095	0.4168	0.5882	0.2381	0.9796
	**6**				0.0372	0.3117	0.1469	0.0680
	**8**					0.3731	0.1441	0.5100
	**10**						0.7412	0.7478
	**16**							0.4097
	**24**							
**IL1ra**		**2**	**4**	**6**	**8**	**10**	**16**	**24**
	**2**		0.8337	0.2886	0.9078	0.6454	0.2457	0.6626
	**4**			0.2485	0.8572	0.6258	0.2486	0.7000
	**6**				0.7178	0.4069	0.6078	0.2248
	**8**					0.6661	0.5646	0.7672
	**10**						0.3647	0.8656
	**16**							0.2865
	**24**							
**MRC1**		**2**	**4**	**6**	**8**	**10**	**16**	**24**
	**2**		0.7488	0.3842	0.0007	0.5648	0.5007	0.8252
	**4**			0.1018	0.0025	0.4226	0.3949	0.5433
	**6**				0.0048	0.8499	0.8391	0.5605
	**8**					0.0003	0.0205	0.0083
	**10**						0.7647	0.7846
	**16**							0.6493
	**24**							
**ARG1**		**2**	**4**	**6**	**8**	**10**	**16**	**24**
	**2**		0.0138	<.0001	<.0001	0.0004	0.0208	0.0085
	**4**			0.0106	0.0020	0.0041	0.4579	0.7186
	**6**				0.0018	0.2438	<.0001	0.0012
	**8**					0.1388	<.0001	<.0001
	**10**						0.0014	0.0156
	**16**							0.1544
	**24**							
**CD86**		**2**	**4**	**6**	**8**	**10**	**16**	**24**
	**2**		0.2042	0.4318	0.9169	0.5731	0.2545	0.0203
	**4**			0.8782	0.3780	0.8207	0.8871	0.4193
	**6**				0.5538	0.9294	0.8339	0.5795
	**8**					0.6455	0.4060	0.1343
	**10**						0.8108	0.6164
	**16**							0.7600
	**24**							
**TGFβ1**		**2**	**4**	**6**	**8**	**10**	**16**	**24**
	**2**		0.7553	0.8404	0.0200	0.8186	0.7694	0.2791
	**4**			0.6795	0.1164	0.9889	0.8582	0.0118
	**6**				0.0406	0.8702	0.9094	0.0356
	**8**					0.2138	0.0431	0.0127
	**10**						0.9236	0.1373
	**16**							0.0771
	**24**							

**Figure 3:**
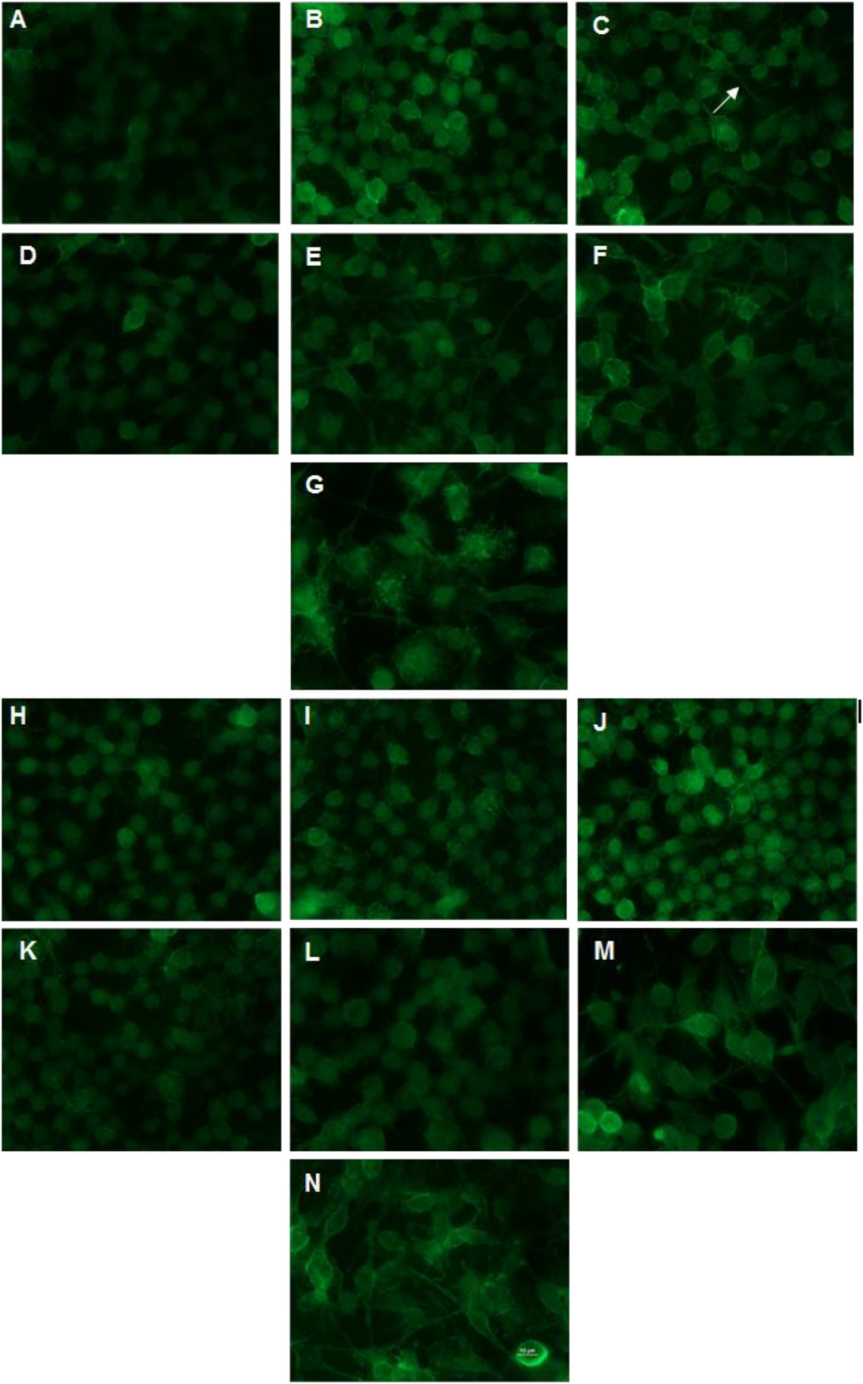
**BV2 cell culture exposed to CD11b immunofluorescent staining over time. A-G** Immunofluorescent staining of no-treatment control cells at 2, 4, 6 8, 10, 16 and 24 h, respectively. **H-N** Staining of cells exposed to the IL-4 treatment at 2, 4, 6, 8 10, 16 and 24 h, respectively. Images were taken with approximately the same cell confluency at a magnification of 60× and show a higher density of processes in the no treatment control compared to the IL-4-treated cells until 16 h. *Arrow* indicates extended processes seen on the untreated BV2 cells at 6 h.

The morphology of the CD11b-positive BV2 cells over this time course was monitored by immunofluorescent staining, shown in Figure 3. Generally, CD11b-positive microglial cells did not differ after treatment with IL-4 in spite of a clear difference in the inflammatory phenotype. We did observe a qualitatively decreased density of extended processes of the BV2 cells in the IL-4-treated groups compared to the no-treatment control groups, but this was not quantifiable (Figure 3).

**Figure 4:**
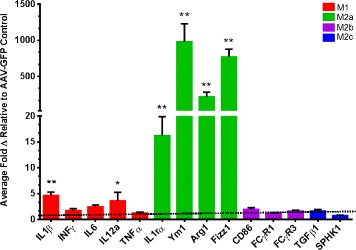
**IL-4 induces an M2a neuroinflammatory phenotype in APP/PS1 transgenic mice.** The figure shows the relative gene expression for genes representative of the M1, M2a, M2b and M2c phenotypes relative to the 18-s endogenous control. Data are shown as the average fold change (± SEM) normalized to the AAV-GFP control. *P < 0.05 between the AAV-GFP control (*N* = 8) and AAV-IL-4 mice (*N* = 6). **P < 0.01 between the AAV-GFP control and AAV-IL-4 mice.

A sandwich ELISA was performed to confirm that the AAV-8 was an effective vehicle for gene delivery resulting in IL-4 expression. Protein extracts taken from the right frontal cortex showed a marked significant increase (*P* = 0.0145) in IL-4 levels (mean 32.2 ng IL-4/mg protein) in the mice receiving AAV-IL-4 in comparison to the AAV-GFP-treated mice (mean 2.4 ng IL-4/mg protein). Figure 4 indicates the neuroinflammatory profile of the AAV-IL-4 mice relative to that of the AAV-GFP controls. Real-time qPCR was performed from RNA extracts from the right hippocampus. Genetic probes were selected based on their role in M1, M2a, M2b and M2c phenotypes identified in peripheral macrophages. As hypothesized, a robust M2a phenotype can be observed as a result of the AAV-IL-4 injection. IL1ra, YM1, ARG1 and FIZZ1 proved to be significantly different from the control. In particular, YM1 and FIZZ1 showed a 700–1,000-fold change. Elevated expression of M1-specific genes can also be identified with significant increases in IL1β and IL12a.

To determine the morphology and activation of microglia in response to the AAV-IL-4 treatment, tissue sections were stained with a β-integrin marker, CD11b. In the hippocampus, Figure 1A and B shows an increased trend in positive CD11b immunostaining. In particular, the DG reveals a positive increase in the positive immunostaining (Figure 1C and D). An arrow in Figure 1C highlights microglial activation around the injection site of the AAV-IL-4 mice. Interestingly, we found increased CD11b staining in the hippocampus of AAV-IL-4 mice, which was statistically significant in the dentate gyrus region (Figure 1E), but we did not see changes in the frontal cortex (Figure 1F).

**Figure 5:**
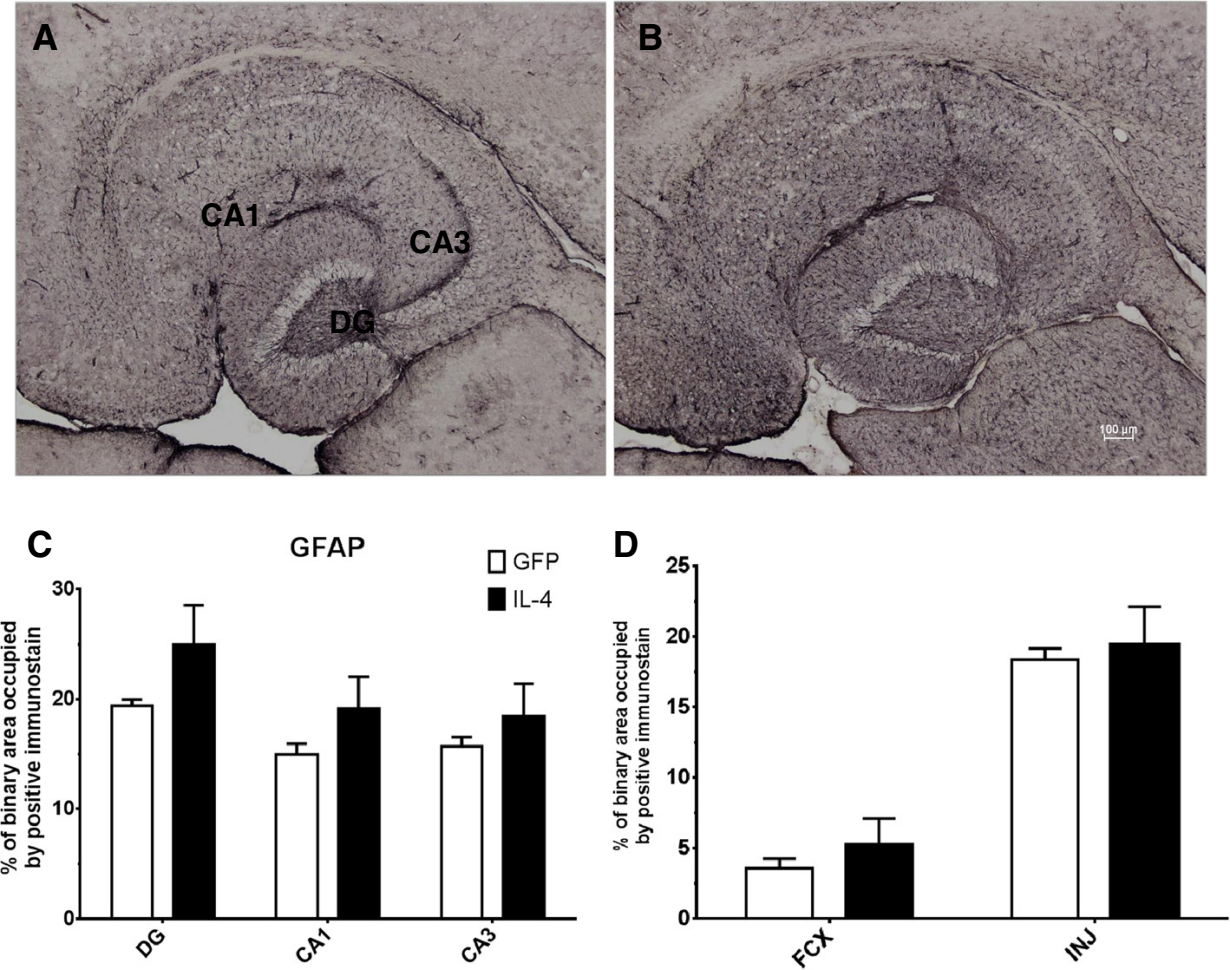
**IL-4 shows increased GFAP immunostaining. A** and **B** GFAP staining of hippocampal sections of the AAV-GFP- and the AAV-IL-4-injected mice, respectively. **C** The percentage of binary area occupied by the positive immunostain in the hippocampus across all samples; **D** uses (*N* = 4) for AAV-IL4 samples because of damage in the frontal cortex of two AAV-IL-4 mice. *P < 0.05 between the AAV-GFP control (*N* = 8) and AAV-IL-4 (*N* = 6).

In addition, immunostaining was conducted with GFAP to assess the effect of the AAV-IL-4 treatment on astrogliosis. Figure 5A and B shows a modest increase in positive immunostaining, exemplified in Figure 5C, but fails to show any significant results. Once again, analysis on the frontal cortex was performed on a sample size of *N* = 4 in the AAV-IL-4 mice, consistent to that of the CD11b stain. It should be noted that an increase in astroglial staining could be observed in the AAV-GFP mice around the hippocampal injection site.

**Figure 6:**
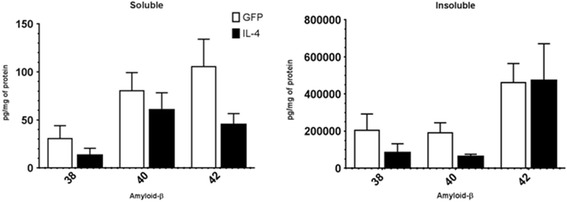
**IL-4 causes a small decrease in Aβ deposition.** This figure shows the relative amyloid-β expression (1–38,1-40,1-42) detected using a Meso Scale Discovery assay (MSD) for the AAV-GFP control and the AAV-IL-4-treated APP/PS1 transgenic mice. No statistically significant difference was seen between the AAV-GFP control and AAV-IL-4 groups, but the results show a decreasing trend (*N* = 8 AAV-GFP and *N* = 6 for AAV-IL-4). Immunostained Aβ plaques with Congo red could not be detected because the mice were only 3 months old. Compact plaques can be seen at 6 months of age.

Aβ depositions in the APP/PS1 transgenic mice were determined biochemically on the MPER soluble and insoluble (formic acid soluble) protein extracts. An MSD ELISA multiplexed assay was used to analyze the concentration of Aβ 1–38, Aβ 1–40 and Aβ 1–42 in the pg/mg of the protein extract (Table 3). Modest decreases are shown in both the Aβ soluble extracts despite no marked significance (Figure 6A). An exception to this is the Aβ1-42 depositions in the insoluble extract. Immunostaining for Aβ and Congo red staining proved to be inconclusive because of the scarce compact plaque formation due to the young age of the survival of the mice. Values for the Aβ species concentrations are given in Table 4.

**Table 3:** **Soluble and insoluble Aβ levels measured by ELISA**

**AAV injection**	**Soluble (pg/μg protein)**			**Insoluble (ng/μg protein)**		
	**Aβ-38**	**Aβ-40**	**Aβ-42**	**Aβ-38**	**Aβ-40**	**Aβ-42**
GFP-AAV	30.6 ± 12.6	80.4 ± 17.7	**105.6 ± 26.8**	**204.6 ± 82.1**	**191.5 ± 62.6**	461.8 ± 96.0
IL-4-AAV	13.2 ± 6.7	60.4 ± 16.3	**45.2 ± 8.6**	**83.0 ± 45.1**	**62.6 ± 12.1**	472.2 ± 181.5

Bold font indicates *P* < 0.05 compared to GFP-AAV of the same time point.

**Table 4:** **Fold change values for RT-PCR in APP/PS1 transgenic mice**

**Gene**	**GFP-AAV**	**IL-4-AAV**
**IL1β**	1.20 ± 0.27	**4.63 ± 0.67**
**INFγ**	1.20 ± 0.39	1.70 ± 0.32
**IL6**	1.33 ± 0.40	2.43 ± 0.38
**IL12a**	1.18 ± 0.31	**3.60 ± 1.64**
**TNFα**	0.74 ± 0.51	1.24 ± 0.15
**IL1ra**	1.41 ± 0.44	**16.24 ± 3.62**
**YM1**	1.90 ± 1.04	**979.43 ± 251.30**
**ARG1**	1.27 ± 0.40	**216.17 ± 65.91**
**FIZZ1**	2.80 ± 1.74	**767.81 ± 108.26**
**CD86**	1.19 ± 0.29	1.91 ± 0.36
**FCγR1**	1.22 ± 0.33	1.23 ± 0.07
**FCγR3**	1.15 ± 0.26	1.61 ± 0.17
**TGFβ1**	1.21 ± 0.30	1.62 ± 0.28
**SPHK1**	1.03 ± 0.10	1.61 ± 0.12

Bold font indicates *P* < 0.05 compared to GFP-AAV of that time point and genotype.

## Discussion

Inflammation and Aβ are suggested to work both independently and synergistically, contributing to the development of AD symptoms and pathological progression [2,17-19]. It remains unknown whether inflammation has a direct or indirect effect on the acceleration of Aβ pathology, with data showing both increased and decreased amyloid deposition [20,21]. The current study aimed to characterize the M2a response stimulated by IL-4 both *in vitro* and *in vivo*. Further, we investigated the effect of an M2a phenotype on Aβ levels in the brain. The time courses of microglial morphology and phenotypic alterations in response to the application of IL-4 applied exogenously were assessed using the immortalized murine microglial cell line, BV2. *In vivo*, IL-4 was delivered to the brain using an AAV vector in the APP/PS1 transgenic mouse model. IL-4 is a typical inducer of an M2a phenotype [11]. An M2a phenotype was indeed induced following IL-4 application using both *in vitro* and *in vivo* approaches. The BV2 microglial cells showed a time-dependent heterogeneous phenotype with the M2a response being the most robust. These results concurred with the *in vivo* study; again, an evident M2a phenotype was induced with depleted Aβ deposits. Unfortunately, due to significant death in the mice receiving the IL-4 AAV, we terminated the study prematurely; thus, our measurement of Aβ deposition was restricted to biochemical outcomes.

IL-4 was selected as our stimulus for an M2a phenotype as it has been shown to be the standard stimulator of an M2a phenotype in peripheral macrophages [11]. Application of IL-4 onto BV2 cells revealed a robust time-dependent increase in M2a phenotypic markers with the vast majority showing a statistically significant peak in expression at 8 h. Overall, phenotypic expression in IL-4-induced BV2 cells strongly validates the gene screening seen in the *in vivo* model. In addition to the exhibition of a robust M2a state, increases in M1 and M2c could also be observed indicating an overall heterogeneous phenotype in response to the IL-4 application. This heterogeneity suggests links between these markers, emphasizing the spectral nature of microglial phenotypes and the potential interplay of the mechanistic actions of downstream pathways. Hence, further investigations are required to decipher these cascades. The time course shows that IL1β is increased in the BV2 cells in direct proportion to the increase in the IL1ra. It is possible that the increase in IL1ra is a homeostatic response to counteract amplified expression of IL1β. IL1β has a diverse range of physiological roles within the CNS [22]; therefore the benefits of complete antagonism to achieve an M1 phenotype could be detrimental to maintaining overall homeostasis within the tissue. In addition, the M2a phenotypic genes ARG1 and MRC1 both peak at 8 h. Future studies may examine other mechanisms by which we can promote an M2a response. For example, IL-13 is also an M2a-promoting cytokine, overlapping with the IL-4 pathway by dimerization of the shared IL-4Rα with IL-13Rα1 to activate a STAT-6 mechanism [23].

Immunofluorescent CD11b was used to visualize the microglial morphology at the sequential time points. CD11b is a microglial surface integrin expressed solely during activation, and it was previously indicated that disruption to homeostasis induces an “amoeboid” retraction of ramified processes, an enlarged cytoplasm, which is an indication of the activated state [24,25]. Extended filopedia can be seen up until the 6-h time point in the no-treatment control, suggesting a maintained resting state as opposed to the retracted and condensed shape compared to that of the IL-4-treated cells demonstrating activation. There is no distinct correlation between the morphology at the time points with the inflammatory profile despite the minor morphological changes discussed.

Investigation of the inflammatory profile from RT-qPCR of the right hippocampus of mice injected with IL-4 expressing AAV indicated a robust M2a phenotype with significant elevations in all inflammatory markers. Extracellular matrix remodeling proteins YM1 and FIZZ1 showed highly amplified levels reaching 500–1,000 fold. IL1ra showed a marked increase, neutralizing the role of IL1β. Congruent to the trend identified in the BV2 cells, the immune profile also exhibited increases in M1 phenotypic genes, specifically IL1β and IL-12a, suggesting a heterogeneous immune profile with chronic exposure to IL-4. Furthermore, this exemplifies the continuum of functionality in which microglia lie. It is evident that elevation of the M1 phenotype has responsive ties to that of the M2a phenotype, as seen in the BV2 cells. Phenotypic heterogeneity was further enforced by Weekman et al. [26], who revealed a mixed phenotype with IFNγ stimulation after 6 months of exposure. Unlike the BV2 cells, elevations of an M2b or M2c could not be observed in the mouse tissue.

CD11b immunohistochemistry is used to visualize activated microglia generating an indication of gliosis in a given tissue area. Astrocytes are also considered to have an immune-regulatory role. GFAP staining visualizes astrogliosis, proliferation of astrocytes in a given area. In this study, the data reveal increases in positive immunostaining in response to the AAV-IL-4 treatment, indicating a responsive proliferation of both cell subsets associated with elevated M2a phenotypic expression. Specifically, staining with CD11b revealed marked, statistically significant positive immunostaining in the dentate gyrus of the hippocampus, suggesting increased microgliosis in this region. Data from the GFAP staining also show increased immunoreactivity, but there was no statistical significance in any region of the hippocampus or frontal cortex. What is particularly intriguing is that these markers of “inflammation” that are presumed to represent proinflammatory responses are actually significantly increased in the presence of the M2a phenotype, which is often considered “antiinflammatory”. This may suggest a re-consideration in the field as to what these immunohistochemical markers functionally represent.

Data on the effect of the M2a phenotype on amyloid deposition were inconclusive. We did find a decreasing trend in Aβ levels in response to the AAV-IL-4 despite no marked statistical significance. This may have been due to the premature termination of the study. IL-4 has been studied in relation to AD pathology with mixed outcomes. Indeed, a study design very similar to the current study used AAV to overexpress IL-4 in the TgCRND8 mouse model. Here, increased amyloid deposition was observed 6 weeks post injection. Similar to the current study, enhanced GFAP and CD11b immunohistochemistry was also observed [13]. IL-4 has also been shown to attenuate Aβ-mediated neuroinflammation in a rat model [27] and promote the clearance of oligomeric Aβ by microglia *in vitro* [28]. The conflicting data highlight the complexities of the neuroinflammatory response and provide the rationale for further studies using better agents to chronically stimulate specific phenotypes.

Understanding the role of inflammation in Alzheimer’s disease is accelerating within the field. Yet, the direction at which to target inflammation remains a hot topic of contention. Overall, the investigation accentuates that the microglial phenotypic continuum is influenced by multitude of dimensions. This study has emphasized that intricate cytokine networks function on a time- and concentration-dependent scale. Cytokine interplay needs to be further investigated in detail to maximize the therapeutic success of immune modulation. Further studies should address the heterogeneous inflammatory profile of the AD brain at different stages of progression across all etiologies and the physiological impacts of aging on microglial ability to retain their function. In addition, the complex communications between the differing glial cells of the CNS should be acknowledged when targeting the immune response. Taken together, research in this field should move toward elucidating a personalized therapeutic approach to treating individual AD patients.

## Conclusions

Our data show that IL-4 induces a robust M2a phenotype in microglial cells in both *in vitro* and *in vivo* models with implicating elevations of M1 phenotypic markers. The M2a inflammatory state is associated with increased microgliosis and astrogliosis as evidenced by increased CD11b and GFAP-positive immunostaining. Additionally, biochemical analysis shows modest reductions in soluble and insoluble Aβ lengths in APP/PS1sw transgenic mice at 3 months of age.

## Abbreviations

AD: Alzheimer’s disease
Aβ: Beta-amyloid
IFNγ: Interferon gamma
IL-1β: Interleukin 1 beta
TNFα: Tumor necrosis factor alpha
IL-12: Interleukin 12
IL-6: Interleukin 6
ARG1: Arginase 1
FcγR 1 and 3: Fc gamma receptor 1 and 3
TGFβ: Transforming growth factor beta
SPHK1: Sphingosine kinase 1
LPS: Lipopolysaccharide
AAV: Adeno-associated virus
SEM: Standard error of the mean
MRC1: Mannose receptor 1
YM1: CHL3, Chitin-like protein
CD86: Cluster of differentiation 86
1× DPBS: Dulbecco’s phosphate-buffered saline
ABC: Advidin-biotin complex
DAB: Diaminobenzidine
CA: Cornu ammonis
DG: Dentate gyrus
FCX: Frontal cortex
MPER: Mammalian protein extraction reagent
BCA: Bicinchonic acid
MSD: Meso-scale discovery
ELISA: Enzyme-linked immunosorbent assay
RT-PCR: Real-time polymerase chain reaction
ANOVA: Analysis of variance
GFAP: Glial fibrillary acidic protein
